# Autophagy in gastrointestinal cancers

**DOI:** 10.3389/fonc.2022.975758

**Published:** 2022-08-26

**Authors:** Bo-Zong Shao, Ning-Li Chai, Yi Yao, Jin-Ping Li, Helen Ka Wai Law, En-Qiang Linghu

**Affiliations:** ^1^ Department of Gastroenterology, General Hospital of the Chinese People’s Liberation Army, Beijing, China; ^2^ Department of Health Technology and Informatics, Faculty of Health and Social Science, The Hong Kong Polytechnic University, Hunghom, Hong Kong SAR, China

**Keywords:** autophagy, gastrointestinal cancer, esophageal cancer, gastric cancer, colorectal cancer

## Abstract

Gastrointestinal cancers are a group of cancers occurred in gastrointestinal tissues with high morbidity and mortality rate. Although numerous studies were conducted on the investigation of gastrointestinal cancers, the real mechanisms haven’t been discovered, and no effective methods of prevention and treatment of gastrointestinal cancers have been developed. Autophagy, a vital catabolic process in organisms, have been proven to participate in various mechanisms and signaling pathways, thus producing a regulatory effect on various diseases. The role of autophagy in gastrointestinal cancers remains unclear due to its high complexity. In this review, firstly, the biological features of autophagy will be introduced. Secondly, the role of autophagy in three popular gastrointestinal cancers, namely esophageal cancer, gastric cancer, and colorectal cancer will be described and discussed by reviewing the related literature. We aimed to bring novel insights in exploring the real mechanisms for gastrointestinal cancers and developing effective and efficient therapeutic methods to treat gastrointestinal cancers.

## Introduction

Gastrointestinal cancers are generally referred to as a group of cancers occurred in the gastrointestinal tissues, including the esophagus, stomach and colon/rectum (1). According to recent studies, it has been reported that each year, 4 million cases of gastrointestinal cancers are diagnosed in the world, and 2.7 million people die from gastrointestinal cancers annually (1–3). In a broad sense, gastrointestinal cancers are comprised of malignant tumors occurred in other digestive system tissues, including liver, small intestine, gallbladder, bile duct and pancreas (4, 5). Based on that knowledge, to keep it accurate and convincing, in our study, we focused on discussing the mechanisms of the three commonly acknowledged kinds of gastrointestinal cancers, including esophageal cancer, gastric cancer, and colorectal cancer. Although there are many different biological features of gastrointestinal cancers, several common risk factors have been discovered, including pro-tumoral genetic mutations, smoking, excessive alcohol intake, western diet, disturbance of gastrointestinal microbiota homeostasis and radioactive stimulation (6–9). In addition, the disturbance of normal gastrointestinal microenvironment is revealed to potentially cause gastrointestinal cancers, such as the pro-tumoral fibrosis and overwhelmingly local or overall inflammatory and immune responses (10–12). Besides those commonly acknowledged risk factors, some disorders have been proven to be closely related to the cause of gastrointestinal cancers. For instance, diabetes has been revealed to be associated with gastrointestinal cancer. Metformin, one of the most well-known anti-hyperglycemic drugs, was shown to reduce the incidence rate of gastrointestinal cancers in diabetic patients (4, 13). Based on that knowledge, the restoration of metabolic homeostasis and prevention of tumor-associated microenvironment formation might serve as potentially effective methods to treat gastrointestinal cancers.

Autophagy has been commonly recognized as a vital metabolic mechanism in organisms (14, 15). It is proven to be effective in degrading and recycling long-term and misfolded proteins, useless organelles under certain stressful conditions such as hypoxia, nutritional deficiency and inflammatory stimulation (16–18). So far, autophagy has been indicated to participate in numerous mechanisms and signaling pathways or cascades, thus regulating various kinds of diseases in many systems (19–22). For example, in digestive system, autophagy has been proven to contribute to the regulation of gastrointestinal microbiota (23–26). Although numerous studies reported and discussed the effect and mechanisms of autophagy in gastrointestinal cancers (27–34), the role of autophagy in gastrointestinal cancers is still not clear, and the real mechanisms remain unclarified. This situation makes it hard to use autophagy to prevent and treat gastrointestinal cancers.

In this paper, we will review related literature on the study of autophagy in three commonly diagnosed gastrointestinal cancers, including esophageal cancer, gastric cancer, and colorectal cancer. We will focus on the introduction and discussion of the controversial role of autophagy in such types of cancers, aiming to provide novel insight in the recognition of autophagy in gastrointestinal cancers, and seeking to develop an effective method to use autophagy to treat gastrointestinal cancers.

## Part I: Biological characteristics of autophagy

The word “autophagy” was initially created in the 1960s by Dr. Christian de Duve, who was awarded the Nobel Prize in 1974 for the discovery of the lysosome as a new specialized membrane-bound organelle in animal cells (35). The word “autophagy” was derived from the Greek roots “auto” (self) and “phagy” (eat). It was referred to as the cellular metabolic processes in which cytoplasmic proteins and certain organelles were “eaten” by itself (to eat it self) (36–38). Since its initial discovery, the mechanisms of autophagy and its roles in cellular catabolic processes and diseases have been widely studied and recognized by researchers. In 2016, Dr. Yoshinori Ohsumi was awarded the Nobel Prize in Medicine or Physiology for his work of investigating the processes of cellular autophagy (39). So far, three types of classic autophagy have been divided based on the difference in mode of cargo delivery to the lysosomal lumen and physiological functions, including microautphagy, chaperone-mediated autophagy and macroautophagy (40–42). Microautophagy is recognized as a non-selective lysosomal process. Degrading proteins and organelles are engulfed *via* the invagination of the lysosomal/vacuolar membranes through microautphagy (43, 44). Chaperone-mediated autophagy is a form of selective autophagy which relies on the recognition of chaperons *via* targeted motif in the degrading proteins and lysosomal chaperons (45, 46). Macroautophagy is the most studied form of autophagy. Macroautophagy is referred to as a metabolic process with the functional unit of double-membraned autophagosomes, which are subsequently fused with lysosomes for further degradation and recycling (47, 48). Besides the classic classification of autophagy, some special forms of selective autophagy have been discovered and studied, including pexophagy, mitophagy, xenophagy and reticulophagy, etc. Those special forms of selective autophagy represent the special forms and functions of autophagy in certain organelles and conditions (49–51).

**Figure 1:**
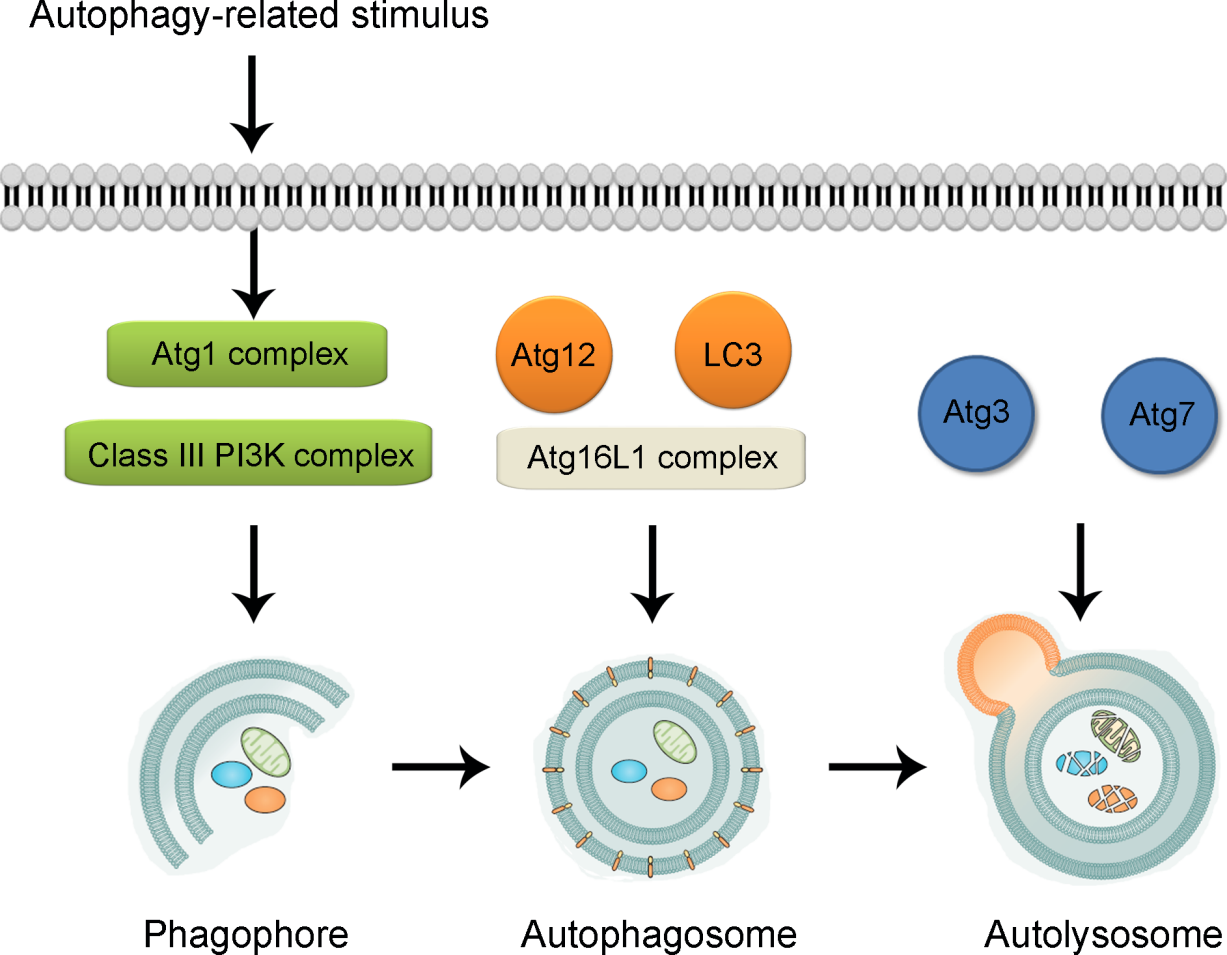
Illustration of biological process of autophagy. Under the challenge of autophagy-related stimulus, autophagy process is triggered through the formation of cup-shaped phagophore participated by Atg1 complex and Class III PI3K complex. With the formation of Atg16L1 complex and assistance of Atg12 and LC3, the bilayer membrane undergoes expansion, elongation and nucleation, which are sequestrated into double-membrane sphere-shaped autophagosomes. With the participation of Atg3 and Atg7, autophagosomes fuse with lysosomes to form the functional autolysosomes. Atg, autophagy-related gene. PI3K, phosphatidylinositol 3-kinase; LC3, light chain 3.

Autophagy-lysosomal system is recognized as one of the two classic protein degrading pathways along with ubiquitin-proteasome system (52, 53). The process of autophagy has been revealed to be mediated by more than 30 autophagy-related genes (Atgs), most of which are proven to be conserved in mammal cells (54, 55). According to our previous study, the induction of autophagy mainly follows two steps (56, 57) (illustrated in **Figure 1**). In the first step, under the challenge of stressful conditions such as starvation and hypoxia, cup-shaped phagophores with lipid bilayer membrane is formed to wrap around substrate materials. The formation of phagophores demands the formation of the Atg1 complex and Class III phosphatidylinositol 3-kinase (PI3K) complex, with Unc-51-like kinase (ULK1, aka Atg1 in yeast), FIP200, Atg13, Atg101 assembly for Atg1 complex and Beclin-1, Atg14, vacuolar protein sorting 15 (VPS15), and VSP34 for PI3K complex (58, 59). After the initiation, the bilayer membrane undergoes expansion, elongation and nucleation, which are sequestrated into double-membrane sphere-shaped autophagosomes. Such process is dependent on the formation of Atg16L1 complex, assembled by Atg5, Atg12 and Atg16L1 (60, 61). In addition, two ubiquitin-like proteins including Atg12 and Atg8 [LC3 (light-chain 3)] also participate the process of autophagosome formation. In the second step, autophagosomes dispose of “coat proteins (LC3-II)” on the surface of the membrane and fuse with lysosomes assisted by Atg3 and Atg7 to form the functional autolysosomes (62, 63). For the regulation of autophagy process, the Class I PI3K-mammalian target of rapamycin (mTOR) is shown as an inhibitory pathway of autophagy through the stimulation of mTOR complex 1 (mTORC1) (64). The Class III PI3K pathway is illustrated as an inductive pathway for autophagy with the formation of Class III PI3K-Beclin-1 complex (65, 66). So far, several kinds of autophagy inducers and inhibitors have been widely used in the modulation of autophagy level in both experimental studies and clinical practice. For instance, rapamycin is widely used to up-regulate the level of autophagy through the inhibition of mTORC1 activation (67, 68). For the inhibition of autophagy, the mechanism of 3-methyladenine (3-MA) for the inhibition of autophagy is through the inhibition of Class III PI3K complex formation (69). In addition, chloroquine is *via* the influence of the acidic environment of lysosomes and bafilomycin A1 is *via* the disturbance of the formation of autolysosomes (70–72).

In the recent few decades, autophagy has been illustrated to participate or regulate numerous diseases in many systems, including atherosclerosis and hypertension (cardiovascular system) (73, 74), diabetes and obesity (metabolic system) (75, 76), ischemic stroke and multiple sclerosis (central nervous system) (77, 78), inflammatory bowel disease and gastritis (digestive system) (23, 79) as well as malignant tumors (80–82). In the treatment of cancers, the interventions to both induce and suppress autophagy have been shown as effective therapies, which indicates the complication of autophagy in the pathogenesis and progression of cancers (83). According to research literature, in the onset of cancers, autophagy can effectively prevent cancer cell formation *via* the correction of pro-tumoral genetic mutations and clearance of mutated cells (84, 85). Conversely, once cancer cells are formed, autophagy may promote cancer cell survival and growth through the autophagy-mediated cellular protection (80, 86). That knowledge reveals the difference between basal autophagy and stimulus-induced autophagy in cancers. The studies also indicate the different roles and effects of autophagy in the different stages of cancer progression.

## Part II: The role of autophagy in gastrointestinal cancers

**Figure 2:**
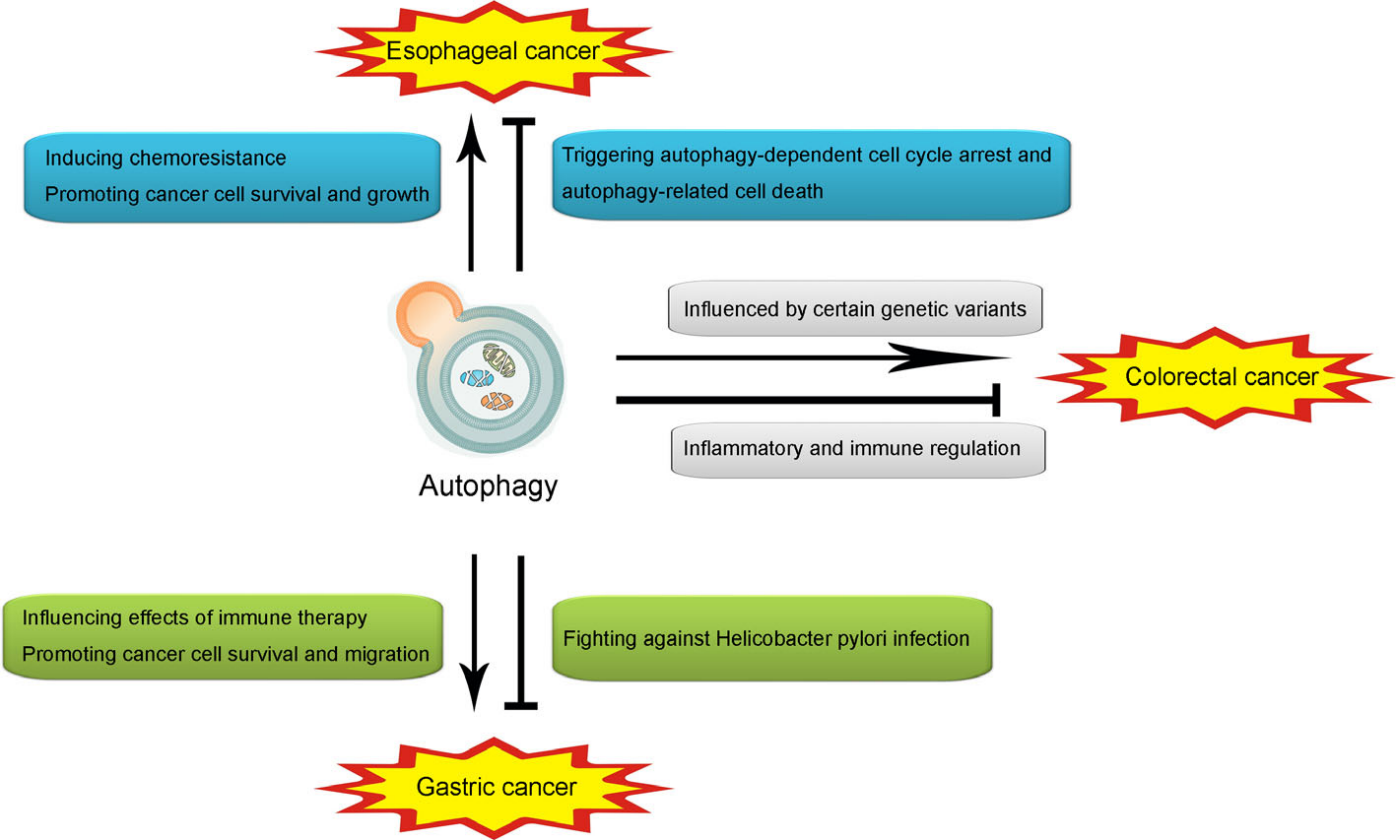
Illustration of the role of autophagy in esophageal cancer, gastric cancer and colorectal cancer. Autophagy may promote esophageal cancer through inducing chemoresistance and promoting cancer cell survival and growth. However, autophagy may also attenuate esophageal cancer through triggering autophagy-dependent cell cycle arrest and autophagy-related cell death. In gastric cancer, autophagy may promote the onset and development through influencing effects of immune therapy. However, autophagy may also attenuate it through fighting against *Helicobacter pylori* infection. In colorectal cancer, autophagy may promote the pathogenesis and progression through the influence of certain genetic variants in autophagy, while producing an attenuative effect through inflammatory and immune regulation.

As discussed above, the role of autophagy is controversial in cancers. Such contradiction also exists in gastrointestinal cancers. In the following contents, the role of autophagy in several popular gastrointestinal cancers, including esophageal cancer, gastric cancer, and colorectal cancer, will be further discussed by reviewing previous related studies (illustrated in **Figure 2**).

### Autophagy in esophageal cancer

Esophageal cancer is a type of malignant tumor that occurs in the esophagus, which is a long and hollow tubular organ ranging from throat to stomach (87, 88). According to recent statistics, although the incidence rates vary from different geographic locations, esophageal cancer is listed as one of the most common causes of cancer deaths all over the world (89, 90). The risk factors of esophageal cancer include Barrett’s esophagus, smoking, alcohol abuse, obesity, achalasia and frequent hot liquid intake, etc. (91, 92). The typical symptoms of esophageal cancer include dysphagia, chest pain, coughing and weight loss (92, 93). However, in some cases, due to its hidden onset, esophageal cancer is hard to be controlled and managed in the early stage. As a result, the research study of pathogenic mechanisms and specific screening markers for esophageal cancer is urgent.

So far, autophagy has been demonstrated to be closely associated with esophageal cancer. Four Atgs including DNAJB1, BNIP1, VAMP7 and TBK1 were shown to act as prognostic signature for the recognition of high- and low-risk groups among people (94). Another Atg signature including (VIM, UFM1, TSC2, SRC, MEFV, CTTN, CFTR and CDKN1A) were demonstrated to contribute to the improvement of the prediction of clinical outcomes in esophageal cancer patients (95). According to a research study, 22 autophagic long-chain noncoding ribonucleic acids were revealed to be highly correlated with the overall survival of esophageal adenocarcinoma patients, thus creating a novel prognostic model for esophageal adenocarcinoma (96).

For the role of autophagy in esophageal cancer, Fang et al. (97) reported that under the treatment of diketopyrrolopyrrole (DPP), autophagy was induced as a self-protective mechanism of esophageal cancer cells from DPP. Autophagy was also shown to mediate microRNA-193b-related chemoresistance on 5-fluorouracil (5-FU) treatment (98). Those findings indicated that the suppression of autophagy-mediated chemoresistance might be a potential strategy for adjuvant chemotherapy in esophageal cancer. In addition, microRNA-498 was shown to suppress esophageal cancer through the inhibition of autophagy and M2-like polarization of macrophages *via* mouse double minute 2 (MDM2)/activating transcription factor 3 (ATF3) signaling pathway (99). Jia et al. (100) reported that Phlorizin, an important member of the dihydrochalcone family derived from sweet tea, could suppress the progress of esophageal cancer through the promotion of cellular apoptosis and inhibit the level of autophagy in esophageal cancer cells. Those effects might lead to antagonizing the Janus kinase-2 (JAK2)/signal transducer and activator of transcription 3 (STAT3) signaling pathway. Furthermore, up-regulation of Beclin-1, a vital member of Atgs, can lead to a more aggressive esophageal squamous cell phenotype and a worse survival prognosis, thus indicating Beclin-1 to be a potential and promising prognostic biomarker and therapeutic target for patients with esophageal squamous cancer (101).

Conversely, some researches have reported the opposite effective of autophagy on esophageal cancer. According to a previous study, silencing autophagy *via* the inhibition of ATP6V1C1 has been shown to serve to enhance radiotherapy resistance in esophageal squamous cell carcinoma (102). In addition, the induction of autophagy *via* certain agents have been reported to trigger autophagy-dependent cell cycle arrest and autophagy-related cell death in cancer cells. For instance, dihydroartemisinin (DHA), the primary active derivative of artemisinin, produced an anti-tumor effect on esophageal cancer cells through the triggering of autophagy-dependent cell cycle arrest at the G2/M phase (103, 104). Therefore, further studies are demanded for the exploration of the role of autophagy in esophageal cancer.

### Autophagy in gastric cancer

Gastric cancer, or stomach cancer, is amongst the most aggressive human malignant tumors all over the world, representing a heavy health burden (105, 106). An estimation of 90%-95% gastric cancers belong to adenocarcinomas. Besides, other types of gastric cancer can start in the stomach, including gastrointestinal stromal tumors (GISTs), neuroendocrine tumors and lymphomas, etc. (107, 108). So far, several risk factors have been commonly recognized, including western diet, obesity, age, gender and bacterial infection (109–111). Notably, *Helicobacter pylori* (*H. pylori*) infection is regarded as an independent risk factor, especially cancers in the lower (distal) part of the stomach (112). It is reported that gastric cancer patients are at higher infectious rate of *H. pylori* than people without gastric cancer (113). Like the situation of esophageal cancer, the prevention and management of gastric cancer in the early stage are difficult because of its latent symptoms in many cases.

In recent studies, the role of autophagy in gastric cancer has been widely investigated. According to many research studies, suppression of the level of autophagy might serve as an effective approach in the treatment of gastric cancer. For instance, the level of autophagy was shown to be associated with programmed cell death-1 (PD-1) with its ligand (PD-L1). Wang et al. (114) reported that inhibition of autophagy could enhance the expression of PD-L1, thus promoting the sensitivity to PD-L1-related immune therapy. In addition, UPR-induced autophagy activation was triggered by Sec62, a membrane protein of the endoplasmic reticulum that facilitated protein transport. Such pathway of autophagy induction was shown to contribute significantly to the metastasis of gastric cancer (115). In another study, Xu et al. (116) demonstrated that c-Jun N-terminal kinase (JNK)/extracellular signal-regulated kinase (ERK)-dependent autophagy was connected to gastric cancer cell survival, and the inhibition of JNK/ERK-dependent autophagy enhanced the Jaspine B derivative-induced gastric cancer cell death through the p62/Keap1/Nrf2 signaling pathway. Furthermore, oncogenic autophagy in gastric cancer cells were demonstrated to be controlled by mucolipin TRP cation channel 1 (MCOLN1), a lysosomal cation channel, *via* the mediation of zinc influx into the cytosol (117).

However, the situation of *H. pylori* infection has been shown to be different. It has been demonstrated that autophagy could protect against *H. pylori* infection (118). The *H. pylori* toxin vacuolating cytotoxin (VacA) and genetic deficiency of autophagy could promote *H. pylori* infection and thus contributing to the incidence of gastric cancer (118, 119). Sustained exposure to *H. pylori* was shown to inhibit autophagy process in gastric epithelial cells at least partly *via* the Nod1-nuclear factor (NF)-κB/mitogen-activated protein kinase (MAPK)-ERK/fork head box O 4 (FOXO4) signaling pathway (120). In addition, the induction of autophagy degrading functions by vitamin D3 could prevent gastric epithelial cells against *H. pylori* infection (121). Based on those findings, to fully take advantage of autophagy in the treatment of gastric cancer, the controversial roles of autophagy should be taken into thorough consideration, especially the effect of autophagy on *H. pylori* infection.

### Autophagy in colorectal cancer

Colorectal cancer is a common malignant tumor of digestive tract, usually occurring at the junction of rectum and left half colon (122). It is considered to be the fourth most common malignant tumor, ranking third at 11% of all malignant tumors diagnosed all over the world (123, 124). The pathological types of colorectal cancer include adenocarcinoma, mucinous adenocarcinoma, squamous carcinoma and undifferentiated carcinoma, etc. The etiology of colorectal cancer is complicated and remains unclear. Currently, it is commonly recognized that the incidence of colorectal cancer is related to smoking, high-fat and low-cellulose diet, intestinal inflammation and other factors (125, 126). As for the occurrence and development mechanism of colorectal cancer, recent studies indicate that the inflammatory immune microenvironment of tumor cell growth plays an important role in such processes. Patients with chronic inflammatory bowel diseases, such as ulcerative colitis, have a significantly increased incidence of colorectal cancer, with enteritis-related colorectal cancer accounting for 6.7% of the total population during a 30-year follow-up period (127). The proportion of patients with ulcerative colitis who developed poor prognosis types of colorectal cancer such as signet ring cell carcinoma or myxoid carcinoma has increased significantly (128–130). Therefore, regulating the over-activation of inflammatory and immune reaction thus controlling the formation of tumor-associated inflammatory and immune microenvironment is vital for the prevention and treatment of colorectal cancer.

As shown in previous research and studies from us and other researchers, autophagy could function in the regulation of inflammatory and immune responses in many inflammation- and immune-related diseases, such as inflammatory bowel disease (IBD) (79, 131–135). Such inflammatory and immune regulating effects have also been revealed by numerous studies in colorectal cancer. For instance, autophagy was shown to couple the environmental signals and metabolic homeostasis to protect lineage and survival integrity of Treg cells, thus preventing tumor resistance and development of inflammatory disorders (136). The induction of mitophagy, a form of selective autophagy, could trigger the anti-tumor adaptive immunity during tumorigenesis (137). In addition, autophagy in intestinal epithelial cells was demonstrated to prevent tumorigenesis *via* the restoration of DNA damage and prevention of cell proliferation and inflammation, while deficiency in autophagy promoted tumor progression of colorectal cancer (138, 139). Induction of autophagy by certain small molecular agents were reported to protect against colitis-associated colorectal cancer *via* suppressing the NLR family pyrin domain containing 3 (NLRP3) inflammasome activation (140). Furthermore, as shown in our previous study, autophagy could attenuate the growth and metastasis of colorectal cancer through the modulation of neutrophil extracellular traps (NETs) and inflammasomes (141). However, another study revealed that the Thr300Ala variant in Atg16L1, one of the vital Atgs, was associated with improved overall survival in human colorectal cancer (142). Those findings indicated that certain mutation of Atgs might produce a therapeutic effect on colorectal cancer.

## Conclusion

In this review, we have introduced and discussed the role of autophagy in three popular gastrointestinal cancers, including esophageal cancer, gastric cancer and colorectal cancer. As discussed, the effects of autophagy on the gastrointestinal cancers are controversial and complex. Although there have been many research studies conducted on this topic, the real mechanisms of autophagy in gastrointestinal cancers remain unclarified. To fully take advantage of autophagy in the treatment of gastrointestinal cancer, further studies are demanded on this topic.

## Author’s contributions

B-ZS, N-LC and YY retrieved concerned literatures and wrote the manuscript. J-PL designed the figures. EL and HL revised the manuscript. All authors contributed to the article and approved the submitted version.

## Conflict of interest

The authors declare that the research was conducted in the absence of any commercial or financial relationships that could be construed as a potential conflict of interest.

## Publisher’s note

All claims expressed in this article are solely those of the authors and do not necessarily represent those of their affiliated organizations, or those of the publisher, the editors and the reviewers. Any product that may be evaluated in this article, or claim that may be made by its manufacturer, is not guaranteed or endorsed by the publisher.
